# On the Various Tests for Saccharine Urine and on the Varieties of Diabetes

**Published:** 1858-09

**Authors:** 


*On the Various Tests for Saccharine Urine, and on the Varieties of Diabetes.* By Dr. A. Becquerel.

Dr. Becquerel draws attention to certain fallacies that may arise in the employment of the potassio-cupric liquid of Barreswil, the solution of Trommers, or caustic potash, as tests for sugar in the urine. The following method, he states, prevents all fallacy: To a measured quantity of urine, say thirty parts, add a smaller quantity of solid acetate of lead in crystals, say two parts; heat being applied, a dirty-white precipitate is at once obtained; this liquid is to be filtered, and the filtrate treated with the sulphate of soda in excess, say four parts. The second mixture is to be again heated; the sulphate of lead is precipitated, and a clear, transparent liquid remains, which contains the sugar, if any was present, the urea, and some saline matter. The potassio-cupric solution is not reduced, nor liquor potassæ turned brown, unless sugar is present in this liquid. If albumen is present in the urine, the acetate of lead carries it down with the other organic matter contained.

After various remarks on the purely chemical aspects of the question, Dr. Becquerel passes to the consideration of diabetes; which he regards either as idiopathic or symptomatic. The former is characterized by the presence of a notable amount of sugar in the urine, which is increased in quantity; there is excessive thirst and hunger, with other morbid phenomena. In the latter the presence of some sugar in the urine is an accessory symptom following upon other diseased conditions; like albuminuria, it is associated with a great variety of diseases. In these cases, the sugar is never very considerable, though it may amount to as much as 25 or 26 per 1000; while in idiopathic diabetes it rises to 60 and even 80 per 1000. In symptomatic diabetes neither the quantity nor the density of the urine is materially increased.

Dr. Becquerel divides the conditions with which symptomatic diabetes may be associated into five categories:

1. Diseases of the brain and cord.
2. Diseases of the liver.
3. Diseases accompanied by dyspnœa.
4. The presence of lactation.
5. Various diseases.

Among nearly two thousand patients, whose urine the author has caused to be examined at the Hôpital de la Pitié, he has found five cases belonging to the first category; they were respectively:

1. A case of myelitis in a woman, aged thirty-seven, who died tetanic, and had sugar constantly in her urine.
2. A case of general paralysis in a woman, aged fifty four, with temporary convulsive affections, during which the urine was saccharine.
3. Amaurotic amblyopia, with a paralytic condition of the lower extremities, in a man, aged fifty-one; urine permanently saccharine.
4. A man, aged sixty-two, closely resembling the last case.
5. A young woman, aged twenty-two, with meningo-cephalitis, during which there were 8 to 12 grammes of sugar per 1000 in the urine. Recovery: five weeks later, return of the same symptoms, when there was no sugar or albumen in the urine. Death ensued, and the diagnosis was confirmed by the autopsy.

Dr. Becqueral reports three cases of liver disease accompanied by diabetes.

1. A man, aged fifty-three, with chronic gastritis and chronic hepatic congestions, had 20 to 28 grammes of sugar per 1000.
2. A man, aged fifty-four, with pulmonary emphysema, and consecutive chronic congestion of the liver; the sugar was detected for six months, and then disappeared.
3. A young man, aged nineteen, with slight enteritis and blennorrhagia (there is no further statement about hepatic disease;) being a sugar refiner, he consumed nearly a kilogramme of sugar (about 1¼lb.) daily. Sugar was found in his urine during the whole time of his stay in the hospital.

Dr. Becquerel expected to find sugar frequently in diseases accompanied by embarrassed breathing, but failed to do so entirely.

He found sugar in the urine of nine women recently delivered, in whom the lacteal secretion was established. It was also met with in the two following cases, which do not come under any of the preceding heads:

1. Female, aged thirty-five, affected with cancer of the neck of the womb, not ulcerated.
2. A man, aged fifty-four, affected with extreme anæmia, the result of poverty.—*L' Union Médicale*, and *British and Foreign Med. Chir. Rev.*, from *Southern Med. and Surg. Journal.*

